# Adaptive Neuro-Fuzzy Inference System for Classification of Background EEG Signals from ESES Patients and Controls

**DOI:** 10.1155/2014/140863

**Published:** 2014-03-25

**Authors:** Zhixian Yang, Yinghua Wang, Gaoxiang Ouyang

**Affiliations:** ^1^Department of Pediatrics, Peking University First Hospital, No. 1 of Xian Men Street, Xicheng District, Beijing 100034, China; ^2^State Key Laboratory of Cognitive Neuroscience and Learning and IDG/McGovern Institute for Brain Research, Beijing Normal University, Beijing 100875, China; ^3^Center for Collaboration and Innovation in Brain and Learning Sciences, Beijing Normal University, Beijing 100875, China

## Abstract

Background electroencephalography (EEG), recorded with scalp electrodes, in children with electrical status epilepticus during slow-wave sleep (ESES) syndrome and control subjects has been analyzed. We considered 10 ESES patients, all right-handed and aged 3–9 years. The 10 control individuals had the same characteristics of the ESES ones but presented a normal EEG. Recordings were undertaken in the awake and relaxed states with their eyes open. The complexity of background EEG was evaluated using the permutation entropy (PE) and sample entropy (SampEn) in combination with the ANOVA test. It can be seen that the entropy measures of EEG are significantly different between the ESES patients and normal control subjects. Then, a classification framework based on entropy measures and adaptive neuro-fuzzy inference system (ANFIS) classifier is proposed to distinguish ESES and normal EEG signals. The results are promising and a classification accuracy of about 89% is achieved.

## 1. Introduction

Encephalopathy with electrical status epilepticus during slow-wave sleep (ESES) syndrome is a condition characterized by continuous spikes and waves occurring during sleep [1]. The recent literature refers to it as “ESES syndrome,” which is an age-related reversible disorder with onset at around 4-5 years of age and a generally favorable course with disappearance at around 10–15 years of age [2], usually associated with variable cognitive and behavioral impairments [3, 4]. The pathophysiological mechanisms and neuropsychological deficits associated with this condition are still poorly understood [5]. Therefore, it is important to identify the ESES patients as early as possible such that the clinician can prescribe the necessary medication to stop its progression.

The electroencephalograph (EEG) signal is a measure of the summed activities of approximately 1–100 million neurons lying in the vicinity of the recording electrode. Since it may provide insight into the functional structure and dynamics of the brain [6], exploration of hidden dynamical structures within EEG signals is of both basic and clinical interest and has attracted more and more attention [7–9]. One can assume that the EEG is a signal containing information about the condition of the brain. We can also accept as working hypothesis that the EEG recorded under resting conditions is representative of the global state of the brain [10, 11]. Then, a plausible working hypothesis is that background EEG corresponding to healthy controls is different from that corresponding to patients with pathologies (e.g., ESES). However, it is currently accepted that a human observer hardly discriminates EEG traces of healthy controls from those of ESES subjects. Quantitative EEG analysis using computational methods can therefore assist in the background EEG characterization. The EEG pattern classification scheme usually includes two major parts: feature extraction and classification.

Various methods have been widely used for feature extraction ranging from traditional linear methods such as Fourier transforms and spectral analysis [12] to nonlinear methods such as Lyapunov exponents [13], correlation dimension [14], and similarity [15, 16]. Due to the complex interconnections between billions of neurons, the recorded EEG signals are complex, nonlinear, nonstationary, and random in nature. Therefore, the classification of EEG signals using nonlinear methods that detect and quantify nonlinear mechanisms and thereby better reflect the characteristics of the EEG signals. Nonlinear features may be able to unearth the hidden complexities existing in the EEG time series. Ferri et al. applied the nonlinear cross-prediction test to assess the dynamic properties of the EEG and showed that ESES, like other types of epileptic EEG activities, seems to reflect highly nonlinear and possibly low-dimensional dynamics, whereas non-ESES waking EEG seems to correspond with linear stochastic dynamics [17]. In the recent years, a series of entropy-based approaches have been widely used since they can quantify the complexity (regularity) of an EEG signal [18, 19]. The entropy of the EEG may act as a reliable indicator of changes in cortical neuronal interactions and truly reflect the intracortical information flow [20], and thus the term “entropy” may be more than merely a statistical measure of EEG patterns, which are well exploited using entropies, and it helps in providing distinguishable variation for normal and abnormal biomedical signals [21, 22]. Abásolo et al. applied the approximate entropy (ApEn) to analyse the EEG background activity of Alzheimer's disease (AD) patients and age-matched controls. They found that ApEn is significantly lower in the AD patients at electrodes P3 and P4 [23]. Then, the spectral entropy (SpecEn) and sample entropy (SampEn) were used to analyse the EEG background activity of AD patients and showed that AD patients have significantly lower SampEn values than control subjects at electrodes P3, P4, O1, and O2 but no differences between AD patients and control subjects' EEGs with SpecEn [24]. Burioka et al. found that the ApEn values of EEG signals in absence epilepsy during seizure-free intervals are very similar to those of healthy subjects, but the EEG signals in absence epilepsy during seizure intervals produce significant lower ApEn values than healthy subjects [25]. In the study by Kannathal et al., ApEn was used to investigate the epileptic seizure detection, where three other entropy-based features were extracted and combined with ApEn for studying normal and epileptic EEG signals [26]. A novel feature extraction method based on ApEn, SampEn, and phase entropy was proposed for diagnosing the epileptic EEG signals and showed that the extracted features with fuzzy classifier are able to differentiate the EEGs with a high accuracy [27]. The high identification accuracy was also reported in the study by Song et al. [28], in which they developed a new scheme of automatic epileptic seizure detection on the basis of SampEn feature extraction.

Recently, Bandt and Pompe proposed the permutation entropy (PE) method to measure the irregularity (complexity) of nonstationary time series [29]. The basic idea is to consider order relations between the values of a time series rather than the values themselves. Compared with ApEn and SampEn [21, 22], the advantages of the PE method are its simplicity, low complexity in computation without further model assumptions, and robustness in the presence of observational and dynamical noise [29–31]. Cao et al. used PE to identify various phases of epileptic activity in the intracranial EEG signals recorded from three patients suffering from intractable epilepsy [32]. Li et al. used PE as a feature to predict the absence seizures in genetic absence epilepsy rats and showed a sharp PE drop after the seizures [33]. It was also found that the PE can better extract the pattern of EEG data for the prediction of absence seizure than the SampEn measure. Nicolaou and Georgiou investigated the use of PE as a feature for automated epileptic seizure detection [34]. Bruzzo et al. applied PE to detect vigilance changes and the preictal phase from scalp EEG in three epileptic patients [35]. These results showed that the EEG during epileptic seizures is characterized by a lower value of PE than the normal EEG. It was found that there is a good separability between the seizure-free phase and the preseizure phase and the changes of PE values during the preseizure phase and seizure onset coincide with changes in vigilance state [35].

In terms of classifiers, lots of methodologies have been proposed and applied to process and discriminate biomedical signals [36–38], such as electromyography [39, 40] and EEG signals [41, 42]. In particular, artificial neural networks have been utilized as the most common method for classifying the EEGs. Moreover, fuzzy set theory plays an important role in dealing with uncertainty when making decisions in medical applications. Therefore, fuzzy sets have attracted the growing attention and interest in data analysis, decision making, pattern recognition, diagnostics, and so forth [43, 44]. Neuro-fuzzy systems are fuzzy systems which use ANNs theory in order to determine their properties (fuzzy sets and fuzzy rules) by processing data samples [45]. A specific approach in neuro-fuzzy development is the adaptive neuro-fuzzy inference system (ANFIS), which has shown significant results in classification of EEG signals. Kannathal et al. proposed a novel classification framework based on entropy measures and ANFIS classifier to distinguish normal and epileptic EEG signals [26]. Güler and Übeyli proposed a new scheme using ANFIS and wavelet transform as the classifier, which can identify five types of EEG signals with a recognition rate greater than 98% [45]. Übeyli proposed a system using Lyapunov exponents of EEG signals and ANFIS as the classifier, which can identify these five types of EEG signals with a recognition rate greater than 99% [46]. In the study by Yildiz et al. [47], a wavelet entropy-ANFIS framework is proposed for classifying a state of vigilance as alert, drowsy, or sleep state on an ongoing EEG recording. A classification accuracy of more than 98% is achieved. These results show that ANFIS has potential in classifying the EEG signals.

In this study, a new approach based on ANFIS employing PE and SampEn measures was presented for classification of background EEG signals from ESES patients and controls. The proposed technique involved training the two ANFIS classifiers to classify the two classes of the EEG signals when PE and SampEn of the EEG signals were used as inputs. The goal was to find a clear differentiation between background EEG corresponding to a sample set of ESES patients and that corresponding to healthy control individuals. The paper is organized as follows.  Section 2 presents a description of the data used in this work and briefly describes the extracted features and classifiers that were used.  Section 3 presents the results obtained. Finally, conclusions are given in Section 4.

## 2. Materials and Methods

### 2.1. EEG Data

The EEG data used in this study consists of two different sets. The first set includes EEG recordings that were collected from 10 right-handed healthy subjects. The subjects were awake and relaxed with their eyes open. 100 16-channel EEG epochs of 8 s duration were selected and cut out from each continuous EEG recording after visual inspection for artifacts, for example, due to muscle activity or eye movements. The second set was obtained from 10 patients with ESES, all right-handed. The data set consists of EEG recordings during wakeful state. Similar to healthy data, noise-free segments are selected from the EEG recordings with ESES patients and used for the analysis. All EEG data were recorded by the Nihon Kohden digital video EEG system from a standard international 10–20-electrode placement (Fp1, Fp2, F3, F4, C3, C4, P3, P4, O1, O2, F7, F8, T3, T4, T5, and T6). They were sampled at a frequency of 500 Hz using a 16-bit analogue-to-digital converter and filtered within a frequency band from 0.5 to 35 Hz.

**Figure 1:**
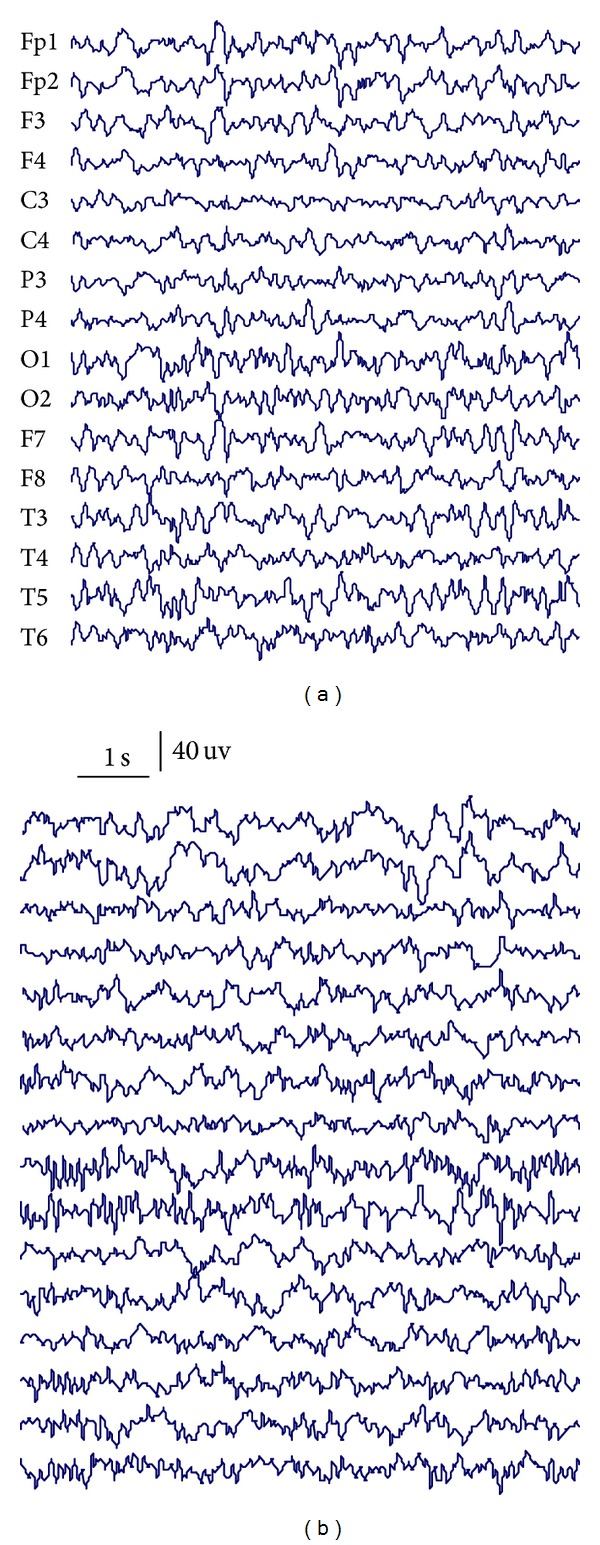
Sample EEG epochs from both ESES patient (a) and control subject (b).

The study protocol had previously been approved by the Ethics Committee of Peking University First Hospital and the patients had signed informed consent that their clinical data might be used and published for research purposes. A summary of the data set is given in Table 1. A sample of EEG epochs from each of the two data sets is plotted in Figure 1.

### 2.2. Sample Entropy

Sample entropy (SampEn) is an algorithm derived from approximate entropy (ApEn) [21]. Introduced by Pincus et al. [48], ApEn is a technique that is useful in determining changing system complexity and it finds application in biomedical research [49]. The first step in computing ApEn of an EEG series {*x*_1_, *x*_2_,…, *x*_*N*_} is to form a sequence of vectors *X*_1_, *X*_2_,…, *X*_*N*−*m*+1_ in *R* ^*m*^, defined by *X*_*i*_ = [*x*_*i*_, *x*_*i*+1_,…, *x*_*t*+*m*−1_] and *X*_*j*_ = [*x*_*j*_, *x*_*j*+1_,…, *x*_*j*+*m*−1_]. Next, we define, for each *i*,

(1) $$\begin{array}{c} C_{i}^{m}\left(r\right)=\frac{i}{N-m+1}\sum_{j=1}\theta \left(r-d\left(X_{i},X_{j}\right)\right), \end{array}$$

where *θ* is the standard Heaviside function and *θ*(*x*) = 1 for *x* > 0, *θ*(*x*) = 0, otherwise; *r* is a tolerance threshold and *d*(*X*_*i*_, *X*_*j*_) is a distance measure defined by

(2) $$\begin{array}{c} d\left(X_{i},X_{j}\right)=\underset{}{\max}\left(\left|x_{i+k-1}-x_{j+k-1}\right|\right),\;k=1,2,\ldots ,m. \end{array}$$

Then, we define *ϕ* ^*m*^(*r*) as

(3) $$\begin{array}{c} \phi^{m}\left(r\right)=\frac{1}{N-m+1}\sum_{i=1}^{N-m+1}\log C_{i}^{m}\left(r\right). \end{array}$$

For fixed *m* and *r*, ApEn is given by the following formula:

(4) $$\begin{array}{c} \text{ApEn}\left(m,r,N\right)=\phi^{m}\left(r\right)-\phi^{m+1}\left(r\right) \end{array}$$

which is basically the logarithmic likelihood that runs of patterns of length *m* that are close (within *r*) will remain close on next incremental comparisons. The more regular the EEG is, the smaller the ApEn will be. The exact value of the ApEn(*m*, *r*, *N*) will depend on three parameters: *N* (length of the time series), *m* (length of sequences to be compared), and *r* (tolerance threshold for accepting matches).

The ApEn specifies a tolerance threshold and so may be better than spectral entropy in the quantification of complexity of EEG recording [50]. The disadvantage of ApEn is that it is heavily dependent on the record length and is often lower than expected for short records. Another disadvantage is that ApEn lacks relative consistency [21]. To overcome the disadvantages of ApEn, a sample entropy (SampEn) was proposed to replace ApEn. By excluding self-matches [21], SampEn reduces the computing time by one-half in comparison with ApEn. Another advantage of SampEn is that it is largely independent of record length and displays relative consistency [51]. The key idea that differentiates SampEn from ApEn is using the correlation sum *C* ^*m*^(*r*) in the entropy definition instead of the *ϕ* ^*m*^(*r*) functions defined in (3)—practically, the position of the log function changes. Thus, Richman and Moorman defined sample entropy as

(5) $$\begin{array}{c} \text{SampEn}\left(m,r,N\right)=\log\frac{C^{m}\left(r\right)}{C^{m+1}\left(r\right)}. \end{array}$$

The choice of input parameters has been discussed by Pincus and Goldberger in [52]. They concluded that, for *m* = 2, values of *r* from 0.1 to 0.25 SD (the standard deviation of the signal) produce good statistical validity of SampEn. In this study, SampEn was estimated with *m* = 2 and *r* = 0.2 × SD of the EEG epoch.

### 2.3. Permutation Entropy

Bandt and Pompe proposed a new permutation method to map a continuous time series onto a symbolic sequence [29], where the statistics of the symbolic sequences was called permutation entropy (PE). PE refers to the local order structure of the time series, which can give a quantitative complexity measure for a dynamical time series [53]. Given a time series {*x*_1_, *x*_2_,…, *x*_*N*_}, an embedding procedure was used to generate *N* − (*m* − 1)*l* vectors *X*_1_, *X*_2_,…, *X*_*N*−(*m*−1)*l*_ defined by *X*_*t*_ = [*x*_*t*_, *x*_*t*+*l*_,…, *x*_*t*+(*m*−1)*l*_] with the embedding dimension *m* and the lag *l*. The vector *X*_*t*_ can be rearranged in an ascending order as [*x*_*t*+(*j*_1_−1)*l*_ ≤ *x*_*t*+(*j*_2_−1)*l*_ … ≤*x*_*t*+(*j*_*m*_−1)*l*_]. For *m* different numbers, there will be *m*! possible order patterns *π*, which are also called permutations. Then, we can count the occurrences of the order pattern *π*_*i*_, which is denoted as *C*(*π*_*i*_), *i* = 1, 2,…*m*!. Its relative frequency is calculated by *p*(*π*) = *C*(*π*)/(*N* − (*m* − 1)*l*). The PE is defined as

(6) $$\begin{array}{c} \text{PE}=-\sum_{m=1}^{m!}p\left(\pi \right)\ln p\left(\pi \right). \end{array}$$

The largest value of PE is log⁡⁡(*m*!), which means that the time series is completely random; the smallest value of PE is zero, indicating that the time series is very regular. More details can be found in [29].

PE calculation depends on the selection of dimension *m* and lag *l*. When *m* is too small (less than 3), the scheme will not work well since there are only a few distinct states for EEG recordings. On the other hand, the length of EEG recording should be larger than *m* in order to achieve a proper differentiation between stochastic and deterministic dynamics [31]. In order to allow every possible order pattern of dimension *m* to occur in a time series of length *N*, the condition *m*!≤*N* − (*m* − 1)*l* must hold. Moreover, *N* ≫ *m*!+(*m* − 1)*l* is required to avoid undersampling [54]. In this study, we therefore choose the dimension *m* = 5 when calculating PE. The lag *l* is referred to as the number of sample points spanned by each section of the vector. The importance of the lag is that it gives the resultant fraction characteristics of the vector. In practice, an autocorrelation function (ACF) of a signal can be employed to automated determination of the lag *l*. An optimal lag can be found at the point where the ACF has firstly decayed to *e* ^−1^ of its peak value [55].

### 2.4. Adaptive Neuro-Fuzzy Inference System

The ANFIS described by Jang [56] is adopted to evaluate the ability and effectiveness of the above entropy measures in classifying the EEG from the ESES patients and control subjects. The ANFIS learns features in the data set and adjusts the system parameters according to a given error criterion. It has been widely used in analysing the biological signals. In order to improve the generalization, ANFIS classifiers are trained with the backpropagation gradient descent method in combination with the least squares method. In this study, two ANFIS classifiers are trained with the backpropagation gradient descent method in combination with the least squares method when 16 features (dimension of the extracted feature vectors; entropy measures from 16-channel EEG) are used as inputs. The samples with target outputs ESES patients and control subjects are given the binary target values of (1, 0) and (0, 1), respectively. The fuzzy rule architecture of the ANFIS classifiers was designed by using a generalized bell-shaped membership function defined as follows:

(7) $$\begin{array}{c} \mu_{ji}\left(x_{i}\right)={\left(1+{\left[\frac{x_{i}-c_{ji}}{a_{ji}}\right]}^{2b_{ji}}\right)}^{-1}, \end{array}$$

where (*a*_*ji*_, *b*_*ji*_, *c*_*ji*_) are adaptable parameters.

Next, two first-order Sugeno-type ANFIS models with 16 inputs and one output are implemented. The first-order Sugeno fuzzy models have rules of the following form:

(8) Ri:IF(x1  is  Ui1⋯  xm  is  Uim)THEN  y  is  gi(x1,…xm)=b0+b1x1+⋯+bmxm,

where *R*_*i*_ is the *i*th rule of the fuzzy system, *x*_*i*_  (*i* = 1,…, *m*) are the inputs to the fuzzy system, and *y* is the output of the fuzzy system; *b*_*i*_  (*i* = 0, 1,…, *m*) are adaptable parameters. The ANFIS output is given by

(9) $$\begin{array}{c} F=\frac{\sum_{j}g_{j}\left(a_{1},a_{2},\ldots ,a_{m}\right)\Pi_{i}\mu_{Uji}\left(a_{i}\right)}{\sum_{j}\Pi_{i}\mu_{Uji}\left(a_{i}\right)}, \end{array}$$

where *μ*_*Uji*_(*a*_*i*_) is the degree of membership of *a*_*i*_  (*i* = 1, 2,…, *m*) to the antecedent linguistic term *U*_*ji*_ for the *i*th rule of the fuzzy system. Each ANFIS classifier is implemented by using the MATLAB software package (MATLAB version 7.0 with fuzzy logic toolbox).

## 3. Results

### 3.1. Entropy Measures of EEG

**Figure 2:**
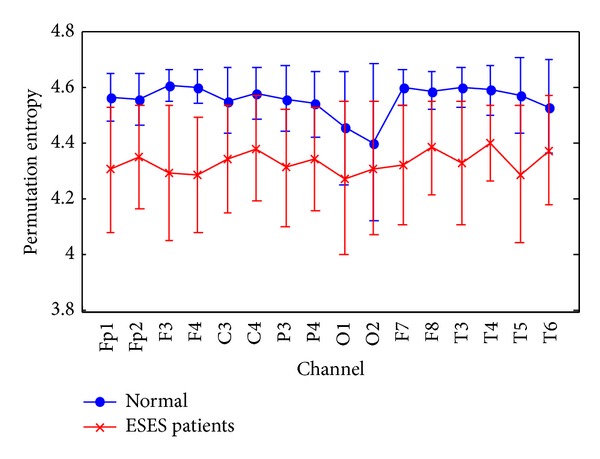
The averaged PE on channel of all EEG recordings, grouped by ESES patients and normal control subjects. Symbols represent the mean values of PE for each group and bars represent the standard error.

EEG epochs from both ESES patients and normal control subjects are investigated in this study. First, PE is applied to analyse the EEG recordings, with *m* = 5, for channels Fp1, Fp2, F3, F4, C3, C4, P3, P4, O1, O2, F7, F8, T3, T4, T5, and T6. The results have been averaged based on all the artefact-free 8 s epochs for each channel. The averaged PEs of all channels are shown in Figure 2. Symbols represent the mean values of PE for each group and bars represent the standard error. It can be found that the PE values of EEG epochs in normal control subjects are much larger than those in ESES patients. The PE values (mean ± SD) for the control subjects and ESES patients and the *P* values of the one-way ANOVA test performed to examine the differences between both groups are summarized in Table 2. It can be seen that ESES patients have significant lower PE values at all 16 electrodes. These results suggest that EEG activity of ESES patients is less complex (more regular) than in a normal control subject. This result supports the view that ESES, like other types of epileptic EEG activity, would reflect low complex and high nonlinear dynamics, whereas non-ESES waking EEG would correspond with high complex dynamics.

**Figure 3:**
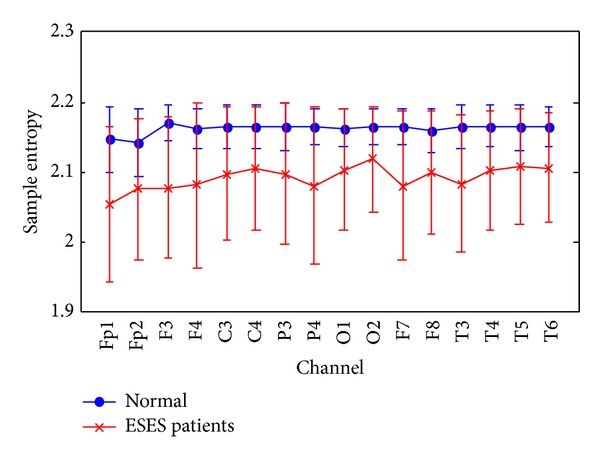
The averaged SampEn on channel of all EEG recordings, grouped by ESES patients and normal control subjects. Symbols represent the mean values of SampEn for each group and bars represent the standard error.

To compare the extracted entropy information of EEG between PE and SampEn methods, SampEn is estimated for channels Fp1, Fp2, F3, F4, C3, C4, P3, P4, O1, O2, F7, F8, T3, T4, T5, and T6 with *m* = 2 and *r* = 0.2  × SD of the original data sequence. The averaged SampEns of all channels are shown in Figure 3. It can be found that the SampEn values of EEG epochs in normal control subjects are also larger than those in ESES patients. However, the SampEn values in control subjects and ESES patients are more overlapped than those of the PE values. Then, the SampEn values (mean ± SD) for the control subjects and ESES patients and the *P* values of the one-way ANOVA test performed to examine the differences between both groups are summarized in Table 3. It can be seen that ESES patients have significant lower SampEn values at all 16 electrodes.

### 3.2. Classification

As shown above, both PE and SampEn values of EEG were significantly different between ESES patients and normal control subjects. The performance of the above measures to discriminate among groups is also evaluated by means of ANFIS classifier, and 10-fold cross-validations are employed to demonstrate the accuracy of classification. First, the ability of the PE in classifying different EEG epochs is evaluated using the ANFIS. Two ANFIS classifiers are trained with the backpropagation gradient descent method in combination with the least squares method when the calculated PE values are used as input. Each of the ANFIS classifiers is trained so that they are likely to be more accurate for one state of EEG signals than the other state. Samples with target outputs sets are given the binary target values of (1, 0) and (0, 1), respectively. Each ANFIS classifier is implemented by using the MATLAB software package (MATLAB version 7.0 with fuzzy logic toolbox). The classification results are illustrated in Table 4. Of 200 EEG epochs in two groups, 178 are classified correctly. Only 4 normal EEG epochs are classified incorrectly by ANFIS as ESES EEG epochs and 18 ESES EEG epochs are classified as normal EEG epochs. The classification accuracy was 89.0%, which is defined as the percentage ratio of the number of epochs correctly classified to the total number of epochs considered for classification.

Then, in order to compare the classification accuracy of PE method with that of the SampEn method, the calculated SampEn values were used as the input data in the ANFIS classifiers, and 10-fold cross-validations were employed to demonstrate the performance of classification. The classification results are listed in Table 5. Of 200 EEG epochs in two groups, 164 were classified correctly. The total classification accuracy was 82.0%. Therefore, it is found that the PE measures can provide a better separability between ESES patients and normal control subjects than the SampEn measures.

## 4. Conclusions

In this study, we have analysed the complexity characteristics in background EEG signals from ESES patients and controls using the entropy measures. Although the background EEG marked was indeed “normal” to standard visual inspection, the proposed methodology based on entropy measures, plus ANOVA statistical test, demonstrates that the background EEG in ESES patients does differ from that in controls. It can be seen that there is a significant increase of the calculated PE and SampEn values of the EEG epochs from ESES patients to control subjects. Then, a new approach based on ANFIS employing entropy measures was presented for classification of background EEG signals from ESES patients and controls. The two ANFIS classifiers were used to classify two classes of EEG epochs when the PE and SampEn of the EEG epochs were used as inputs. The experimental results showed that the classification accuracy, 89%, based on the PE measures is much higher than that with the SampEn measures, 82%. These results suggest that the proposed ANFIS combined with PE measures might be a potential tool to classify the background EEG from ESES patients and normal control subjects. Our next goal is to confirm the results presented here in a much larger clinical cohort of ESES patients.

## Figures and Tables

**Table 1:** Summary of the EEG data.

	Set 1	Set 2
Subjects	10 healthy subjects	10 ESES patients
Age	3–9 years 4 males and 6 females	3–9 years 4 males and 6 females
Patient's state	Awake and eyes open (normal)	Awake and eyes open (no spikes)
Number of epochs	100	100
Epoch duration (s)	8	8

**Table 2:** The average PE values (mean ± SD) of the EEGs for the normal control subjects and ESES patients for all channels.

Electrode	Normal subjects	ESES patients	*P* value
Fp1	4.563 ± 0.088	4.304 ± 0.224	*P* < 0.05
Fp2	4.555 ± 0.089	4.349 ± 0.184	*P* < 0.05
F3	4.606 ± 0.055	4.292 ± 0.240	*P* < 0.05
F4	4.600 ± 0.060	4.286 ± 0.206	*P* < 0.05
C3	4.550 ± 0.116	4.341 ± 0.192	*P* < 0.05
C4	4.579 ± 0.092	4.381 ± 0.190	*P* < 0.05
P3	4.556 ± 0.117	4.311 ± 0.212	*P* < 0.05
P4	4.539 ± 0.116	4.343 ± 0.183	*P* < 0.05
O1	4.454 ± 0.204	4.272 ± 0.273	*P* < 0.05
O2	4.402 ± 0.278	4.308 ± 0.237	*P* < 0.05
F7	4.595 ± 0.064	4.321 ± 0.215	*P* < 0.05
F8	4.585 ± 0.067	4.382 ± 0.168	*P* < 0.05
T3	4.598 ± 0.072	4.331 ± 0.220	*P* < 0.05
T4	4.588 ± 0.090	4.402 ± 0.135	*P* < 0.05
T5	4.571 ± 0.135	4.289 ± 0.248	*P* < 0.05
T6	4.529 ± 0.167	4.374 ± 0.195	*P* < 0.05

**Table 3:** The average SampEn values (mean ± SD) of the EEGs for the normal control subjects and ESES patients for all channels.

Electrode	Normal subjects	ESES patients	*P* value
Fp1	2.147 ± 0.045	2.055 ± 0.111	*P* < 0.05
Fp2	2.144 ± 0.048	2.077 ± 0.101	*P* < 0.05
F3	2.171 ± 0.026	2.078 ± 0.101	*P* < 0.05
F4	2.163 ± 0.029	2.082 ± 0.118	*P* < 0.05
C3	2.166 ± 0.032	2.098 ± 0.095	*P* < 0.05
C4	2.166 ± 0.031	2.107 ± 0.088	*P* < 0.05
P3	2.166 ± 0.034	2.098 ± 0.100	*P* < 0.05
P4	2.165 ± 0.025	2.081 ± 0.111	*P* < 0.05
O1	2.163 ± 0.027	2.103 ± 0.087	*P* < 0.05
O2	2.165 ± 0.025	2.119 ± 0.101	*P* < 0.05
F7	2.165 ± 0.026	2.081 ± 0.106	*P* < 0.05
F8	2.161 ± 0.031	2.099 ± 0.088	*P* < 0.05
T3	2.165 ± 0.032	2.083 ± 0.098	*P* < 0.05
T4	2.166 ± 0.030	2.102 ± 0.085	*P* < 0.05
T5	2.164 ± 0.032	2.109 ± 0.083	*P* < 0.05
T6	2.166 ± 0.029	2.107 ± 0.077	*P* < 0.05

**Table 4:** Classification results with PE measure.

Desired result	Output result	
	ESES patients	Normal subjects
ESES patients	96	4
Normal subjects	18	82

**Table 5:** Classification results with SampEn measure.

Desired result	Output result	
	ESES patients	Normal subjects
ESES patients	92	8
Normal subjects	28	72
